# Differences in treatment choices between prostate cancer patients using a decision aid and patients receiving care as usual: results from a randomized controlled trial

**DOI:** 10.1007/s00345-021-03782-7

**Published:** 2021-07-17

**Authors:** Romy E. D. Lamers, Maarten Cuypers, Marieke de Vries, Lonneke V. van de Poll-Franse, J. L. H. Ruud Bosch, Paul J. M. Kil

**Affiliations:** 1 Department of Urology, University Medical Center, Utrecht, The Netherlands; 2 Department of Primary and Community Care, Radboud Institute for Health Sciences, Radboud University Medical Center, Geert Grooteplein Zuid 10, 6525 Nijmegen, The Netherlands; 3 Institute for Computing and Information Sciences (iCIS) and Social and Cultural Psychology, Behavioural Science Institute, Radboud University, Mercator I, Toernooiveld 216, 6525 Nijmegen, The Netherlands; 4 Department of Research, Netherlands Comprehensive Cancer Organisation, Utrecht, The Netherlands; 5 Department of Medical and Clinical Psychology, CoRPS-Center of Research on Psychology in Somatic Diseases, Tilburg University, Tilburg, The Netherlands; 6 Division of Psychosocial Research and Epidemiology, Netherlands Cancer Institute, Amsterdam, The Netherlands; 7 University Medical Cancer Center Utrecht, Heidelberglaan 100, 3584 Utrecht, The Netherlands; 8 Andros Clinics, Mr. E.N. van Kleffensstraat 5, 6842 CV Arnhem, The Netherlands

**Keywords:** Decision aid, Prostate cancer, Treatment decision making, Shared decision making, Localized prostate cancer

## Abstract

**Objective:**

To determine whether or not decision aid (DA) use influences treatment decisions in patients with low and intermediate risk prostate cancer (PC).

**Patients and methods:**

In a cluster randomized controlled trial, patients were randomized to either DA use (DA group) or no DA use (control group). Between 2014 and 2016, newly diagnosed patients with low or intermediate risk PC were recruited in 18 hospitals in the Netherlands. DA users had access to a web-based DA that provided general PC information, PC-treatment information, and values clarification exercises to elicit personal preferences towards the treatment options. Control group patients received care as usual. Differences in treatment choice were analysed using multilevel logistic regressions. Differences in eligible treatment options between groups were compared using Pearson Chi-square tests.

**Results:**

Informed consent was given by 382 patients (DA group *N* = 273, control group *N *= 109). Questionnaire response rate was 88% (*N* = 336). Active surveillance (AS) was an option for 38%, radical prostatectomy (RP) for 98%, external beam radiotherapy (EBRT) for 88%, and brachytherapy (BT) for 79% of patients. DA users received AS significantly more often than control group. Patients (29 vs 16%, *p* = 0.01), whereas the latter more often chose BT (29 vs 18%, *p* < 0.01). No differences were found between groups regarding RP and EBRT. DA users who were not eligible for AS, received surgery more often compared to the control group (53 vs 35%, *p* = 0.01). Patient and disease characteristics were evenly distributed between groups.

**Conclusion:**

DA-using PC patients chose the AS treatment option more often than non-DA-using patients did.

**Supplementary Information:**

The online version contains supplementary material available at 10.1007/s00345-021-03782-7.

## Introduction

Prostate cancer (PC) is the most frequent malignancy in European men, and incidence rates are rising [1]. The treatment for low and intermediate risk PC often comprises multiple options with comparable oncology outcomes; active surveillance (AS), radical prostatectomy (RP), external beam radiotherapy (EBRT), and brachytherapy (BT) [2]. However, each treatment option entails specific risks and complications that may negatively impact quality of life. Given the variety of benefit and harm profiles and the lack of strong treatment recommendations in current guidelines, localized PC-treatment decision making is highly preference-sensitive and requires adequate shared decision making to find the “best” treatment option for each individual [2, 3].

Decision aids (DAs) support this decision-making process. Previous research has shown that DA users have more accurate risk perceptions and make better-informed and value-based choices than non-DA-users [4–6]. Furthermore, there is evidence that DA use may affect treatment decisions in favour of less invasive and conservative therapies [4, 6]. However, it is unclear whether these results also apply PC decision making as earlier studies were at high risk of bias or did not compare all relevant treatment options [7–14]. Given these uncertainties, we tested the effect of a newly-developed preference-sensitive web-based DA on treatment choice within a cluster randomized controlled trial (CRCT) [15, 16].

## Patients and methods

Between August 2014 and July 2016, newly diagnosed patients with low or intermediate risk PC (EAU/ESTRO criteria) and eligible for at least two different treatments (AS, RP, EBRT, and/or BT) were recruited in 18 hospitals in the Netherlands [17]. Eligible treatments were determined by consent in interdisciplinary team meetings. Exclusion criteria were cognitive impairment, insufficient understanding of the Dutch language, not having an internet connection, or being too ill at the time of the study [15].

### Methods

In a two-armed pragmatic CRCT, hospitals were clustered, meaning that all included patients from a participating hospital were enrolled in the same study group. Participating hospitals could therefore provide the same type of care to all of their patients, making a CRCT less prone to contamination bias [15, 18]. The hospitals were pre-randomized to either ‘usual care’ (control group) or ‘usual care + DA’ (DA group) by a statistician not involved in the study and blind to the identity of the hospitals [19].

Eligible patients were invited to participate by their urologist immediately after diagnosis. Urologists from both groups provided written information and an informed consent form in both groups. At this point, the urologist indicated the eligible treatment options together with their version of the informed consent form. After a diagnosis, but before a treatment decision had been made, patients in the intervention group received access to an online DA. Patients in the control group received decisional counselling as usual. Patients from both groups completed online questionnaires (or paper on request) after the decision was finalized but before treatment onset [15, 20]. The full study protocol was reviewed by the regional medical ethics board who waived the need for formal ethical approval. The study was pre-registered in the Dutch trial register (NTR-4432) and the study protocol has been published separately [15].

### Decision aid

The DA offered stepwise guidance through the decision process, depending on the possible treatment options (in Dutch: www.prostaat.keuzehulp.nl). First, general information about PC and treatment options is provided (benefits and risks, success rates, and complication rates according to the current guidelines and literature) [2]. Next, the DA offers stepwise trade-offs between active surveillance (if eligible) and active treatment (surgery or radiotherapy) first and between surgery and radiotherapy (EBRT and BT) next. Values clarification exercises (VCEs)/statements are presented at each step to elicit patient preferences. Patients can indicate for each set of statements the strength of their preference towards one of the alternatives in the trade-off (e.g. ‘If treatment might be unnecessary, I would rather wait’ vs ‘I prefer treatment, even if it might be unnecessary’). Appendix 1 shows a screenshot of the DA with VCEs concerning the decision trade-off ‘surgery versus radiotherapy’. The development and content of the DA has been described in detail elsewhere [21].

### Outcome measures

The primary outcome measure for this study was received treatment. AS was not defined in the study protocol, however, in the Netherlands, PRIAS criteria (inclusion criteria and follow up schedules) are being followed [22]. For participants whose treatment could not be verified in their medical records, we analysed treatments as reported in the questionnaires. Offered eligible treatment options were obtained from the informed consent forms completed by the urologists.

Standard sociodemographic and clinical information was obtained from the patients’ informed consent forms (date of birth, date of diagnosis) and questionnaires (PSA level, Gleason score, marital status, occupation, and education).

### Statistical analyses

For continuous data, we presented descriptive statistics as means with standard deviations (SD). We reported frequencies and percentages for categorical data. Between group differences were examined using independent samples *t* tests for continuous variables and Chi-square analyses for categorical variables. Fisher’s exact test was used when expected count in cells was less than five.

Because of the multilevel structure of our data and the hospital-level randomization, we performed multilevel logistic regressions to assess treatment differences between groups. Marital status, level of education, PSA level (dichotomized to ≤ 10 μg/l and 10.1–20 μg/l), and Gleason score (6 or 7) were used in our model as fixed effects at the person level. Treatment was randomized at hospital level and added as fixed effect in the relevant analysis; odds ratios (OR) and 95% confidence intervals were reported.

All statistical analyses were conducted using SPSS version 24 (Statistical Package for Social Sciences, Chicago, IL, USA). A *p* value < 0.05 was considered statistically significant.

## Results

Three hundred and eighty-two patients gave informed consent (DA group *N* = 273, control group *N* = 109), of which complete treatment information (eligible and received) was available for 249 DA patients (91%, missing *N* = 24) and for 107 control patients (98%, missing *N* = 2). The questionnaire was completed by = 336 patients (response rate 88%; Appendix 2).

Non-responders were younger than responders (mean = 65.3 vs mean = 62.9, *p* = 0.01). There was no difference in number of offered treatment options between responders and non-responders (*p* = 0.45). For non-responders, no information was available concerning PSA levels and Gleason scores. For 23%, two eligible treatment options were offered, for 49% three options were offered, and for 28% all four treatment options were offered (Table 1).

**Table 1:** Baseline demographics and clinical characteristics of responders *N* = 336

Characteristics	DA group *N* = (%)	Control group *N* = (%)	Total *N* = (%)	*p*
Age at informed consent in years, mean (SD)	64.9 (6.0)	66.3 (5.7)	65.3 (5.9)	0.06
Marital status				
Married/living together	208 (89%)	87 (87%)	295 (88%)	0.54
Other	27 (11%)	13 (13%)	41 (12%)	
Education				
Low	76 (33%)	36 (36%)	112 (34%)	0.40
Medium	54 (23%)	28 (28%)	82 (25%)	
High	101 (44%)	36 (36%)	137 (41%)	
Gleason score				
6	156 (61%)	46 (70%)	202 (63%)	0.25
7	97 (39%)	2 (30%)	117 (37%)	
PSA level				
≤ 10.0 μg/l	207 (81%)	73 (79%)	280 (80%)	0.65
10.1–20.0 μg/l	49 (19%)	20 (21%)	69 (20%)	
Number of eligible treatments				
2	49 (21%)	25 (28%)	74 (23%)	0.51
3	115 (50%)	42 (46%)	157 (49%)	
4	65 (29%)	24 (26%)	89 (28%)	
Offered/eligible treatment options^a^				
AS	102 (39)	36 (36)	138 (38)	0.72
RP	259 (98)	96 (97)	355 (98)	0.45
EBRT	232 (88)	89 (90)	321 (88)	0.70
BT	214 (81)	66 (73)	288 (79)	0.19
Hospital				
1	11 (5%)			
2	1 (1%)			
3	46 (19%)			
4	28 (12%)			
5	13 (6%)			
6	17 (7%)			
7	64 (27%)			
8	35 (15%)			
9	20 (8%)			
10		6 (6%)		
11		18 (18%)		
12		9 (9%)		
13		9 (9%)		
14		23 (23%)		
15		8 (8%)		
16		20 (20%)		
17		8 (8%)		
18		0 (0%)		

Because of missing values, numbers do not always add up to 336

^a^Percentages add to more than 100% because patients were offered multiple treatment options

*SD* standard deviation, *AS* active surveillance, *RP* radical prostatectomy, *EBRT* external beam radiotherapy, *BT* brachytherapy

### Offered/eligible treatment options

We found no differences in offered treatment options between the groups. The majority (98% for both groups) were offered RP (Table 1). AS was offered to 39% in the DA group and to 36% in the control group (*p* = *0*.72), EBRT to 88% and 90% (*p* = 0.70), respectively, and BT to 81% and 73% (*p* = 0.19), respectively.

### Received treatment

Received treatment analysis was performed only for patients who were eligible for that particular treatment. DA-group patients more often chose AS compared to patients in the control group [*N* = 71 (29%) vs *N* = 17 (16%), respectively*,* OR = 3.7, 95% CI 1.33–10.50*, p* = 0.01; Table 2]. Patients in the DA group received BT less often than non-DA-users (*N* = 44 (18%) vs *N* = 31 (29%) respectively, OR = 0.22, 95% CI 0.10–0.47, *p* < 0.001). We found no differences between groups in proportions of patients receiving RP and EBRT.

**Table 2:** Received treatment

	DA group *N* (% column)	Control group *N* (% column)	Total *N* (% column)	Exp (*β*)/OR (95% CI)^a^	*p*^a^
Received treatment					
AS	71 (29)	17 (16)	88 (25)	3.7 (1.33–10.50)	0.01
RP	103 (41)	32 (30)	135 (38)	2.2 (0.96–4.96)	0.06
EBRT	20 (8)	15 (14)	35 (10)	0.67 (0.24–1.9)	0.46
BT	44 (18)	31 (29)	75 (21)	0.22 (0.10–0.47)	< 0.001
Missing/unknown	11 (4)	12 (11)	23 (6)		
Total	249 (100)	107 (100)	356 (100)		

^a^Multilevel regression analyses, marital status, level of education, PSA level (dichotomized to ≤ 10 μg/l and 10.1–20 μg/l), and Gleason score (6 or 7) were used as fixed effects at the person level

Analyses include only patients who were eligible for the selected treatment

*OR* odds ratio, *CI* 95% confidence interval for exp (*β*)/odds ratio, *AS* active surveillance, *RP* radical prostatectomy, *EBRT* external beam radiotherapy, *BT* brachytherapy

### Post-hoc analysis

Of the patients who were eligible for AS, 71/102 patients pursued AS in the DA group and 17/36 patients pursued AS in the control group (70 vs 47%, *p* = 0.01, respectively), as shown in Table 3. This implies that some patients were eligible for AS but still received active treatment: 26 (25%, missing *N* = 5) DA-group patients and 19 (53%) control-group patients chose this option (data extracted from Table 3).

**Table 3:** Received treatment by AS eligibility

	DA group *N* (% column)	Control group *N* (% column)	Total *N* (% column)	*p*
Eligible for AS				
Received treatment AS	71 (70)	17 (47)	88 (64)	0.01
Received treatment RP	17 (16)	11 (31)	28 (20)	0.09
Received treatment EBRT	2 (2)	1 (3)	3 (2)	1.00
Received treatment BT	7 (7)	7 (19)	14 (10)	0.05
Missing/unknown	5 (5)	0 (0)	5 (4)	
Total	102 (100)	36 (100)	138 (100)	
Not eligible for AS				
Received treatment AS	4 (3)	1 (2)	5 (2)	1.00
Received treatment RP	86 (53)	22 (35)	108 (48)	0.01
Received treatment EBRT	19 (12)	14 (22)	33 (15)	0.06
Received treatment BT	40 (24)	24 (38)	64 (28)	0.07
Missing/unknown	13 (8)	2 (3)	15 (7)	
Total	162 (100)	63 (100)	225 (100)	

*AS* active surveillance, *RP* radical prostatectomy, *EBRT* external beam radiotherapy, *BT* brachytherapy

In the DA group, 17/26 patients pursued RP although AS was an eligible treatment option; in the control group, this was the case for 11/19 patients (65 vs 58%, *p* = 0.96, respectively). BT was chosen more often in the control group than in the DA group when active treatment was preferred over AS [7/19 (37%) vs 7/26 (27%) *p* = 0.34, respectively], (data extracted from Table 3).

Finally, DA patients who were not eligible for AS more often chose RP than control patients (53 vs 35%, *p* = 0.01, respectively; Table 3).

## Discussion

This study tested differences in PC-treatment choices between patients who used a DA versus patients receiving standard counselling. Our findings showed an effect from DA use. Patients who used a DA more often chose AS when that was available as option, and preferred RP as active treatment over radiotherapeutic alternatives.

By developing a DA that supported both active and conservative treatment options, our study is the first to show that also among PC patients DA use may increase preference for conservative treatment. This finding implicates that the use of a DA may reduce overtreatment when AS is available as option. As a result, side effects of active treatment may be postponed or even prevented. These results are therefore highly clinically relevant since side effects of prostate cancer treatment can negatively influence quality of life [23–26]. Our results also implicate that our DA provides sufficient information about AS to make DA patients confident to pursue AS. These results are in line with other studies showing that DA use results in more conservative than invasive treatment options [4, 6].

Our results contrast to earlier studies in which no strong effects from DA use on PC-treatment choice were found [7, 9, 13, 14]. Systematic reviews concluded that AS was often not included, not well defined, or was even included as watchful waiting, which may be an explanation for the current findings regarding AS [12, 13]. Finding no statistically significant difference for RP between groups, conforms with the current literature [7, 9, 13, 14].

Another main finding in the current study is that DA-use patients pursued BT less often in comparison to non-DA-use patients. A comparable Dutch study showed more BT after DA use in comparison to usual care, however, that study did not include AS [12]. Even when excluding patients eligible for AS, BT was still opted less among DA users (24 vs 38%), although these differences were not statistically significant (*p* = 0.07, Table 3, post-hoc analyses).

Furthermore, we determined which treatment options were offered to patients after diagnosis, thereby providing insight into decision counselling in clinical practice. 38% of patients were eligible for AS; this proportion is in line with other Dutch studies [27]. Nonetheless, in the present study, 25% of all patients pursued BT. In an American study conducted in the same patient population and period of time as our study, only 10% of the patient population chose BT [28]. Therefore, differences in clinical practice for low and intermediate PC between countries should be interpreted in the light of large national variation and potential cultural differences.

Different from other studies, we were able to stratify patients’ received treatment options by eligibility for AS. Our results showed more AS in DA users among those eligible for AS, and surprisingly, also more RP among those not eligible for AS compared to the control group. So, if patients were eligible for AS, then DA users chose AS more often than non-users did. However, if AS was not an option, DA users chose the most invasive option more often than control-group patients did. Possibly, DA use may reassure patients to choose between extremes. Another explanation may be the bias of counselling by an urologist only, which may lead to more RP instead of radiotherapy.

It, however, remained remarkable to find that a substantial number of patients suitable for AS still preferred active treatment over AS. Fagerlin et al. previously described this tendency towards active treatment: few people can imagine standing by and doing nothing after a cancer diagnosis, regardless of the risks [29]. Possibly, the patients who prefer active treatment over AS are more neurotic/anxious, as neuroticism is associated with PC-specific anxiety [30]. Consequently, these patients’ characteristics should be investigated during PC counselling and information provision should be tailored to this trait. Additionally, AS specific level of information provision satisfaction and AS specific knowledge should be investigated in this group to learn more about these findings.

The main limitation of our study was the imbalanced patient enrolment between groups leading to low control group numbers. Possibly, control-group physicians were not as motivated as DA-group physicians to include patients as they felt they had nothing novel to offer to the patient. We are aware that some important results may be missed due to smaller sample sizes, with emphasis on the post-hoc analysis. However, none of the patient or clinical characteristics were different between study arms, so the lower patient recruitment in the control arm led only to a power reduction and not to a selection bias. Also across individual hospitals inclusion rates varied from *N* = 0 to *N* = 64. All hospitals received the same instructions, and were all visited regularly, and no systematic reason for these differences could be determined. The current results should be interpreted appropriately regarding generalization to other countries considering national variation and potential cultural differences.

The major strength of this study is incorporation of all four treatment options, including AS. Another strength is the cluster randomized design, which reduced the risk of contamination of usual care with components of the DA. The advantage of this design was the relatively natural fit of the DA in clinical practice.

## Conclusions

DA use during treatment decision making for low and intermediate risk prostate cancer may affect treatment decisions. Compared to the control group, our results show more AS and less BT in the DA group. No differences in RP and EBRT proportions were found between groups.

## Supplementary Information

Below is the link to the electronic supplementary material.

Supplementary file1 (PDF 259 kb)

Supplementary file2 (PDF 10 kb)

## Declarations

### Research involving human and animal participants

All procedures performed in studies involving human participants were in accordance with the ethical standards of the institutional and/or national research committee and with the 1964 Helsinki declaration and its later amendments or comparable ethical standards.
