# Supplementary figures and images for: Differences in treatment choices between prostate cancer patients using a decision aid and patients receiving care as usual: results from a randomized controlled trial
Source: World J Urol. 2021 Jul 17;39(12):4327–33. doi: 10.1007/s00345-021-03782-7 (PMC8602175; doi:10.1007/s00345-021-03782-7)

## Appendix B

### Enrolment numbers and flowchart

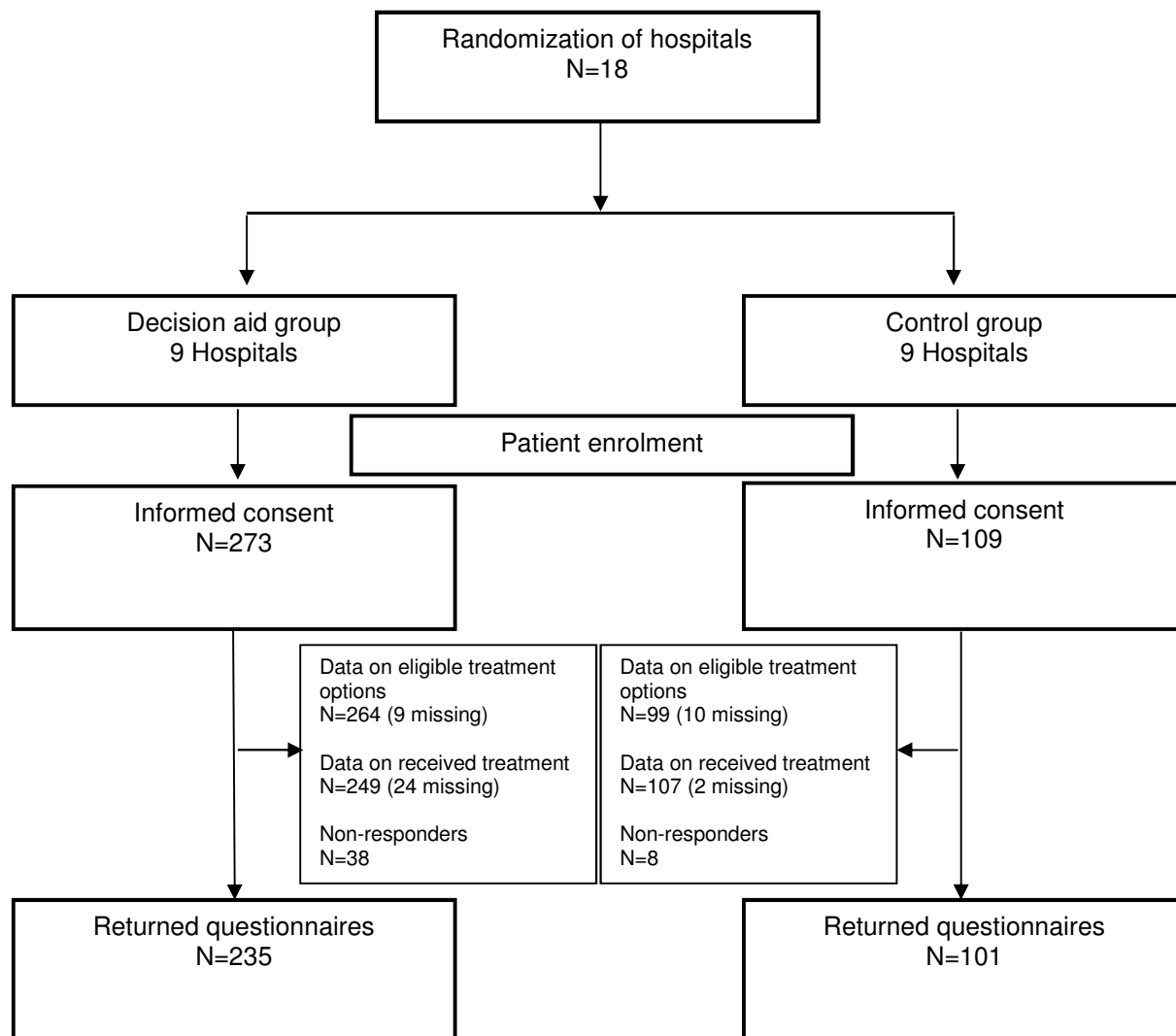

Supplement: Supplementary file 1 — Supplementary file1 (PDF 259 kb) [file 345_2021_3782_MOESM1_ESM.pdf]
