# Supplementary material for: Differences in treatment choices between prostate cancer patients using a decision aid and patients receiving care as usual: results from a randomized controlled trial
Source: World J Urol. 2021 Jul 17;39(12):4327–33. doi: 10.1007/s00345-021-03782-7 (PMC8602175; doi:10.1007/s00345-021-03782-7)
Supplement: Supplementary file 2 — Supplementary file2 (PDF 10 kb) [file 345_2021_3782_MOESM2_ESM.pdf]

Appendix A

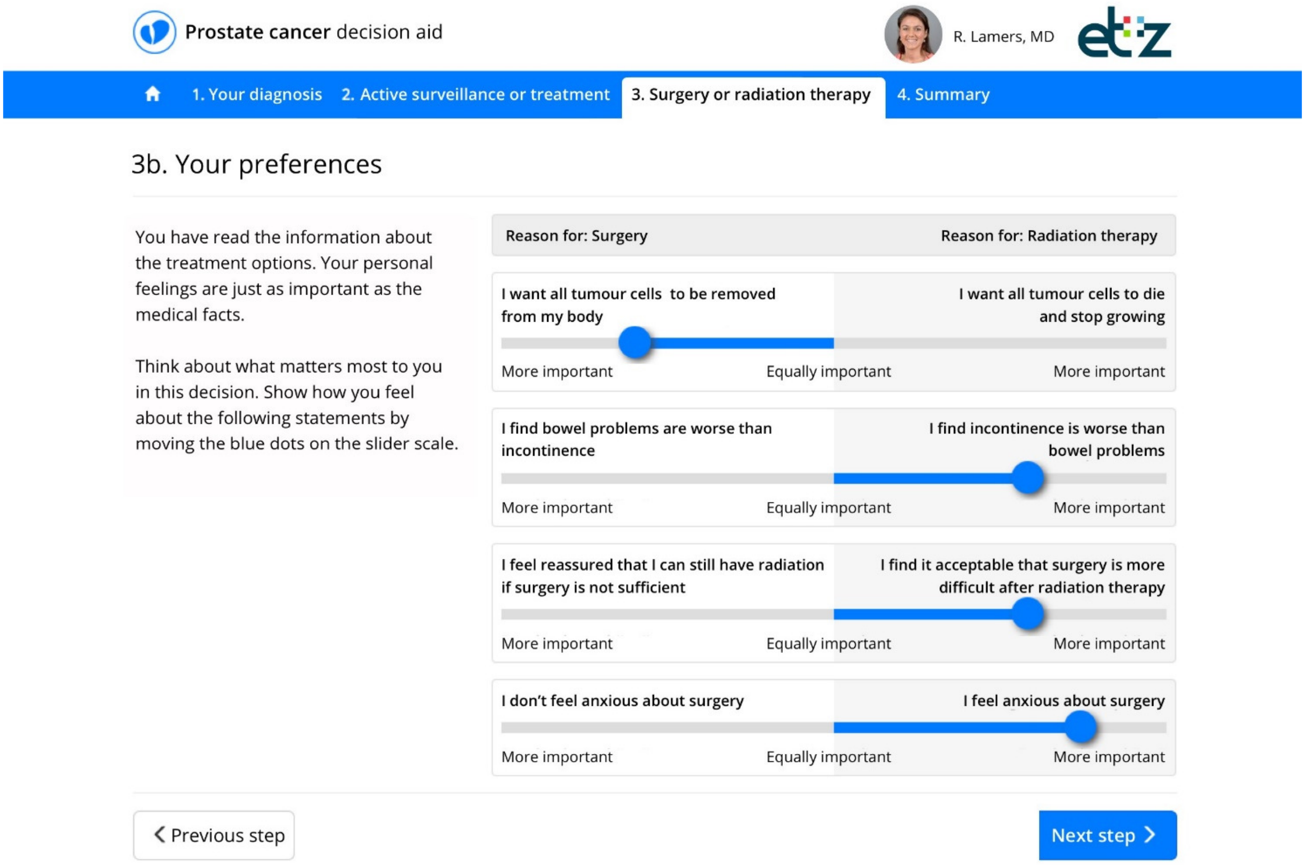
Prostate cancer decision aid
R. Lamers, MD
1. Your diagnosis
2. Active surveillance or treatment
3. Surgery or radiation therapy
4. Summary
3b. Your preferences
You have read the information about the treatment options. Your personal feelings are just as important as the medical facts.
Think about what matters most to you in this decision. Show how you feel about the following statements by moving the blue dots on the slider scale.
Reason for: Surgery
Reason for: Radiation therapy
I want all tumour cells to be removed from my body
I want all tumour cells to die and stop growing
More important
Equally important
More important
I find bowel problems are worse than incontinence
I find incontinence is worse than bowel problems
More important
Equally important
More important
I feel reassured that I can still have radiation if surgery is not sufficient
I find it acceptable that surgery is more difficult after radiation therapy
More important
Equally important
More important
I don't feel anxious about surgery
I feel anxious about surgery
More important
Equally important
More important
Previous step
Next step
